# Health Challenges in Everyday Life of Nigerians in Guangzhou City, China

**DOI:** 10.1007/s12134-023-01013-z

**Published:** 2023-03-03

**Authors:** Kudus Oluwatoyin Adebayo

**Affiliations:** 1 Institute of African Studies, University of Ibadan, Ibadan, Nigeria; 2 African Centre for Migration and Society, University of Witwatersrand, Johannesburg, South Africa

**Keywords:** Africans in China, Healthcare barriers, Medical return migration, Migrant health, Social discrimination of health

## Abstract

The presence of Africans in Chinese cities has made their healthcare-related issues an expanding area of interest. However, previous studies have not thoroughly explored how Africans live through health problems. This article explores the taken for granted aspect using the analytical frameworks of migration as a social determinant of health and phenomenological sociology. Based on interviews with 37 Nigerians in Guangzhou city, it describes how health and illnesses are lived and the ways that language barrier, cost of health care, immigration status and racism and discrimination intertwine with quotidian occurrences to shape the experiences of health challenges. Migrant networks and community structure provided critical assistance, but the context of labour circumstances and undocumentedness can overstretch these critical sources of support. The article exposes how the broader context of being and living in China determine how Africans experience health challenges in Chinese cities.

## Introduction

In 2017, the global population of international migrants was 258 million, a 49% increase when compared with the figure of 2000, which stood at only 173 million (UN DESA, 2017). The health-related consequences of the increase in the population of migrants led the World Health Organisation (World Health Organisation, 2019) to release a draft global action plan which sets priorities for meeting their needs. This move has become necessary as evidence coalesces on the power of migration to impact the health of mobile peoples (Arnold et al., 2014; Castañeda et al., 2015; Fleischman et al., 2015; Zimmerman et al., 2011). Health challenges may arise at departure, during transit or upon arrival at destination. In destination countries, in particular, migrants face multi-dimensional barriers to healthcare (Biswas et al., 2011; Czapka & Sagbakken, 2016; Ransford et al., 2010), with vulnerabilities (Derose et al., 2007; Quesada, 2012), deportability fears (Fleischman et al., 2015) and poor integration impacting their experiences of those barriers (Czapka & Sagbakken, 2016). The barriers lead to reduced access to healthcare (Biswas et al., 2011; Boateng et al., 2012; Czapka & Sagbakken, 2016; Green et al., 2006), vulnerabilities and inequalities in health (Benach et al., 2011).

China has become an important destination for African migrants^1^ (Marfaing, 2019). Attracted by China’s rising position in the ‘globalised economy of manufacture goods’ (Fioratta, 2019), the majority of Africans going to China have, in the last two decades, migrated to the highly commercial centre of Guangzhou in Guangdong Province (Bertoncello & Bredeloup, 2007; Bodomo, 2010; Gordon et al., 2017). Between 2000 and 2007, hotel records indicate that the number of African overnight stayers in Guangzhou increased annually from 6300 to 60,400 (Li et al., 2009). A recent estimate indicates that as many as 22,000 Africans may be residing in Guangzhou city alone (Haugen, 2019), the majority of them being men who engage in trade (Bodomo & Pajancic, 2015; Tu Huynh, 2015). The desire of these Africans to participate in China’s cheap manufacturing economy through transnational trade is no doubt linked to the broader expansion and solidification of China’s economic and political relations with African countries (Braun & Haugen, 2021; Lyons et al., 2013; Mathews & Yang, 2012; Park, 2009; Pieke, 2011; Yang & Altman, 2011).

In cities like Guangzhou, however, the majority of African migrants are transient migrants (Castillo, 2014), implying that a lot of them do not stay for too long. Nevertheless, many have settled down and established families there (Adebayo & Omololu, 2020; Joseph et al., 2017). Among the small population that stay put for a longer period, studies on the integration shows that most of them feel unsettled and express ambivalence in the host society (Adams, 2016; Castillo, 2014). Also, only a negligible number acquiring local language skills and more desiring to return to their countries of origin at some point (Li et al., 2009; Zhou et al., 2016). Settling for a long term is made even worse because many Africans become visa overstayers and forced to live as undocumented after 1 month of entering China (Haugen, 2019; Huang, 2019; Lan, 2017). There is also a problem of widespread racial and stereotypical perception of Africans and black people in China, especially the romance between African men and Chinese women and Afro-Chinese children (Bork-Hüffer & Yuan-Ihle, 2014; Cheng, 2011; T. Liu & Deng, 2020; Pfafman et al., 2015; Sautman, 1994; Wing-Fai, 2015), which likely contributes to the lack of coherent and systematic pathway to citizenship for settled Africans.

Expectedly, the rise in the presence of Africans in China has made their healthcare-related issues an expanding area of interest to researchers. Most of the research on the health context of their presence focuses on the question of accessibility and barriers to health care, mainly in Guangzhou. For instance, Lin et al., (2015a, b) showed that cost, law, language and culture are sources of healthcare barriers among Africans. Also, African patients have a low level of interpersonal trust in Chinese physicians (McLaughlin et al., 2015). Moreover, there is limited knowledge about the diseases affecting Africans in China and language translation services are not available in the hospitals to cater to African clients (Hall et al., 2014; Lin et al., 2015a, b.

Furthermore, the problem of undocumentedness is prevalent in the African community (Huang, 2019; Lan, 2017). Undocumented Africans are afraid to visit the hospital, including African women who are into sex work (Davis et al., 2016). Besides, when compared with internal Chinese migrants, Africans are disadvantaged in many respects, especially in the area of language and visa issues (Bork-Hüffer, 2016).

However, previous studies did not explore how healthcare challenges are lived and grounded in the everyday experiences of African migrants in China. Specifically, there is a limited rigorous attempt to understand how the intertwining of macro-structural factors and realities of the everyday complicate migrants’ experiences of health problems. Based on data obtained from Nigerians residing in Guangzhou city, this article seeks to address this critical knowledge gap. It contends that the experiences of health challenges do not occur in isolation but in conjunction with factors embedded in the ‘migration condition’ itself. This migration condition comprises not only the macro-structural factors but also the everyday circumstances of being a/an Nigerian/African migrant in China.

The article responds to the growing call for a theoretically sound approach that recognises the role of the migration condition in migrant health (Castañeda et al., 2015; Davies et al. 2006; Fleischman et al., 2015). In China especially, the outbreak of COVID-19 and the almost immediate racial discrimination that Africans became exposed to in Chinese cities such as Guangzhou (Adebayo, 2022; Castillo & Amoah, 2020) invites a critical exploration of how health challenges are contextually produced as shaped by the condition of living as a migrant in a destination country. Thus, instead of check-listing the factors causing healthcare challenges, this contribution exposes the social processes behind those factors, making it possible to advance a comprehensive understanding of healthcare challenges in Africa’s migrant community in China. After the introduction, the theoretical perspective of the study would be explained. The theoretical section would be followed by a description of the methodology, after which the findings are presented. The next two sections present a discussion of the salient findings and conclusion.

## Migration as a Social Determinant of Health in Everyday Life: a Framework

Migration as a social determinant of health (MSDH) originates from the thinking that migrants are distinctively vulnerable to poor health and exclusion due to their particular circumstances as migrants (Castañeda et al., 2015; Derose et al., 2007). Whereas migrants may arrive at their destination in good health, thus living up to the thesis of the ‘healthy migrant effect’ in migration health literature (Fennelly, 2007; Markides & Rote, 2019), MSDH takes seriously the manifest decline in the health of migrants that result from context of life as a foreigner in a destination country. MSDH contends that the social condition of being a migrant disproportionately exposes people to situations that have potentially harmful implications for their health. That is:

Being an immigrant limits behavioural choices and, indeed, often directly impacts and significantly alters the effects of other social positioning, such as race/ethnicity, gender, or socioeconomic status, because it places individuals in ambiguous and often hostile relationships to the state and its institutions, including health services. (Castañeda et al., 2015, p. 378)

MSDH stresses the role of social structures, policies and institutions in determining the health outcomes of migrants (Castañeda et al., 2015; World Health Organisation, 2019; Zimmerman et al., 2011). It is about understanding ‘how social and institutional contexts shape individuals’ lives and how factors such as employment, housing and living conditions, access to food and social services and legal status are consequential for well-being’ of migrants (Castañeda et al., 2015, p. 376). Rather than casting migration as a secondary process in the explanation of migrant health, MSDH frames migration as a determinant of health in its own right (Davies et al., 2006). Health challenges, according to this perspective, cannot be reduced to individual behavioural or cultural factors but must be seen as a consequence of broader social processes, that is the ‘health effects of social structures’ (Castañeda et al., 2015, p. 377).

However, there is a need to ground MSDH in the everyday reality of migrants. Grounding MSDH in experience allows us to understand how migrants feel, think about and interpret the health challenges they encounter. Phenomenological sociology is instructive in this regard. Traced to the phenomenology of Edmund Husserl and the reflections of Alfred Schutz in sociology, phenomenological sociology accommodates people’s ‘subjective stock of knowledge’ as socially derived and must be taken account of in the description of reality (Eberle, 2014). Grounding MSDH in experience is vital for understanding how health challenges are lived.

In Guangzhou, studies showed that the organisation of health systems, the problem of illegalisation and the racial regime are remarkable for migrant health dynamics. For example, as the flow of Africans placed new demands on China, the latter is struggling to adjust (Haugen, 2015; Pang & Yuan, 2013) and is yet to take concrete steps towards migrant integration or reforms that would improve the access of foreigners to healthcare. The *hukou* system in China is ‘an official residence status that restricts Chinese citizens’ access to public services to their place of birth’ (Todrys & Amon, 2009, as cited in Zimmerman et al., 2011, p. 4). The health insurance scheme under the system makes health care accessible and cheap for locals, but it excludes foreigners (Davis et al., 2016; Hall et al., 2014; Lin et al., 2015a, b; McLaughlin et al., 2015). More so, China’s strategy to provide primary health care for all by 2020 does not pay attention to the health needs of migrants, even though the policy reform intends to provide primary health care as a form of ‘public service’ (Frontiers of Medicine in China, 2010).

Moreover, the problem of illegalisation in China affects Africans disproportionately and impacts their access to public space and institutions. In cities like Guangzhou, where Africans have a significant presence, the immigration rules have become stricter and more restrictive (Bork-Hüffer & Yuan-Ihle, 2014; Haugen, 2019). Majority of those who initially arrived on a 30-days business visa are unable to renew their stay. Many of them become overstayers, leading to a rise in the problem of undocumentedness in the African community (Haugen, 2012; Lan, 2017). The tightening rules also gave leeway to the police to clampdown on the ‘omnipresent Africans’ (Haugen, 2019), thus resulting in spatial entrapment and immobility (Haugen, 2012). For African migrants, this situation is transforming Guangzhou into a possible space of vulnerability (Schultz, 2014).

Furthermore, the prevailing racial regime affects the presence of Africans and shapes their everyday experience in China. Racialised Othering features in African-Chinese interaction (Liang & Le Billon, 2018). The racialisation contributes to the construction of Africans as womanising, sexually promiscuous disease carriers. This perception is historically situated as evidenced by on-campus anti-African protests and fighting on university campuses between African and Chinese students from 1979 to late 1980s (Cheng, 2011; Sautman, 1994). The culturally embedded nature of the racialisation and its reinforcement through media representations and internal Chinese health promotion strategies are well documented (Cheng, 2011; Hood, 2013). It also contributes to their stereotypical perception as posing a public health threat (Lin, Brown, Yu, et al., 2015; C. Liu, 2015; Zhou et al., 2016).

Departing from the previous approach to the study of health challenges among Africans in China, therefore, this article grounds the social determinant of migrant health in lived experiences with a focus on Nigerians in Guangzhou city. It departs from the linear analysis that had dominated the studies on health challenges of Africans in China and instead expanded on the intricacies of macro-structural factors and the challenging health struggles of the everyday in a migrant community.

## Materials and Methods

### Study Design

The study was exploratory and employed qualitative approaches to collect data from Nigerians in Guangzhou, China. The research from which the data for this article derives involved a total of 69 participants (52 Nigerians and 17 Chinese). However, only the data from 37 Nigerian migrants were considered in this article owing to their extensive discussion of the theme of health challenges (see Table 1). Qualitative approaches have proved appropriate and productive for generating contextual understanding of the health-related issues affecting African migrants in China (Davis et al., 2016; Lin et al., 2015a, b; McLaughlin et al., 2015). The approach is also particularly useful given the study’s interest in grounding health challenges in the day to day experiences of the participants.

**Table 1:** Sociodemographic information of participants

Description	Frequency (*N* = 37)	Percentage (100%)
Sex		
Male	30	81
Female	7	19
Age		
25–34	13	35
35–44	16	43
45–54	8	22
Ethnicity		
Igbo	25	68
Yoruba	8	22
Hausa	2	5
Others	2	5
Marital status		
Married	22	59
Single	13	35
Separated	2	5
Education		
Secondary	12	32
Tertiary	18	49
Not known	7	19
Travel experience		
Yes	11	30
No	26	70
Length of stay		
< 12 months	2	5
1–3 years	11	30
4–6 years	7	19
7–10 years	11	30
> 10 years	6	16
Occupation		
Trading/Business	19	51
Market agent	4	11
Student/Teaching	3	8
Freight Services	3	8
Others	8	22
Visa status		
Valid	25	68
Expired	10	27
Not known	2	5

The study area, Guangzhou, is in Guangdong Province, with a population of around 13 million people (Liang & Le Billon, 2018). It is a major commercial hub that plays host to manufacturing industries, a factor that contributed to the concentration of Africans there (Cornelissen & Mine, 2018), starting from the 1990s and intensified during the 2000s (Bertoncello & Bredeloup, 2007; Li et al. 2008). While Africans of varied nationalities are in Guangzhou, Nigerians easily surpassed others as the most populous group (Bodomo & Pajancic, 2015; Haugen, 2012).

### Data Collection

Data was collected for the larger study that was conducted in Guangzhou in 2017 using a mix of interview approaches—in-depth interview (IDI), key informant interview (KII) and life history (LH)—with participants selected using purposive and snowballing techniques. However, we reached thematic saturation at 37 Nigerian participants, the point when no new information related to experiences of health challenges was being discussed (see Vasileiou et al., 2018). These extracted participants covered 31 IDIs drawn from the general migrant population, four community leaders who took part in KIIs and two LH interviews with long-terms stayers. The array of data collection techniques across different categories of the Nigerian migrant population made it possible to explore experiences of health challenges from multiple perspectives. As shown in Table 1, 30 of the participants (i.e. 81%) were male, and the majority (or 43%) fall within the age bracket of 35 to 44 years. They were overwhelmingly from the Igbo ethnic group, married and almost half of them have some tertiary level education. This demographic is different from the observed pattern in which more of the Africans in the city were unmarried men (Bodomo & Pajancic, 2015). Furthermore, for 26 (70%) participants, China was the only country they have ever visited. The majority have lived in China for more than a year, while six participants have spent 10 years and above. In terms of occupation, trade/business is the most common means of livelihood, but it is normal for migrants to pursue more than one economic activity in Guangzhou. Finally, the majority said they had valid visas at the time of the study.^2^

In response to questions about their access to and experiences of health care services in Guangzhou, participants provided information on a range of issues that shape their healthcare challenges. Interviews were conducted using a mixture of English language and Pidgin English, which are spoken widely by Nigerians. The interviewer introduced himself as a doctoral student from a well-known university in Nigeria. On average, each interview lasted for 60 min. During transcription, aspects of the interviews in the Pidgin English were translated into the standard English language. Interviews took place mainly in shops and markets or other places preferred by the participants.

### Ethical Consideration

The study was reviewed and approved by the Social Science and Humanities Ethics Review Committee of the University of Ibadan. Participants were duly informed about the purpose of the study, and they gave verbal consent. As a cultural insider, some Nigerian participants freely shared their experiences but some were suspicious of my intentions for the same reason (see Adebayo and Njoku, 2023). Nonetheless, safety was a concern for some of the participants, especially those without proper documentation. Allowing them to ask as many questions as possible before commencing the interviews mitigated the safety fears. Also, interviews were conducted where participants were most comfortable. Based on their individual interview experiences, some of them later assisted in facilitating a snowball process which made it possible to reach other participants. The real names of the participants were not used in reporting the findings of the study to ensure anonymity and protect their privacy.

### Data Analysis

After transcribing the audio interviews into texts, the researcher transferred the transcript into a qualitative data analysis software. The interpretative phenomenological analysis (IPA) was adopted to make sense of the data. This approach is appropriate because it enabled the researcher to take into account the ‘personalities, prior life experiences and motivations’ of the participants as well as individual subjective meanings of their experiences (Smith & Osborn, 2004, p. 219). The IPA has been found especially useful by scholars exploring how varied migrant populations think about their lived experiences and make sense of specific health-related experiences in different contexts (Abdulkadir et al., 2019; Griffin et al., 2022; Stuhlhofer, 2021).

On first reading, preliminary codes were generated. These initial codes were read repeatedly to generate additional codes as necessary, and once coding was finalised, similar codes were identified and grouped under master codes which became the themes used to organise the experiences of participants. The IPA guided the analysis process interview narratives were read to extract participants’ subjective sense making and interpretations around the challenges they faced, health-wise, in their day to day life as migrants in the city. The themes are presented in the next section and discussed in more detail using summaries and paraphrasing. Where necessary, supporting verbatim quotations from the participants are presented.

## Findings

### Health and Illness Among Nigerian Migrants

In exploring the health challenges of Nigerians, participants discussed their perceived health status and illness pattern in Guangzhou city based on both direct and indirect experiences. Most of the Nigerians interviewed for the study perceived themselves as having good health, with fewer people complaining of poor health. However, this may not reflect the actual situation on the ground.

According to some participants, including community leaders, many Nigerian migrants do not visit the hospital for routine check-ups or minor illnesses unless there was an emergency. A community leader stated that ‘Nigerians…don’t take care of themselves…and when things get very difficult or serious, there’s nothing you can do; by that time, it will be too late’ (KII 1/Male/48 years). Another community leader reiterated the same view when he said that ‘our people [Nigerians] are not good at going for a medical check-up; they only go [to the hospital] when they are sick; when it is an emergency’ (KII 2/Male/45 years). This suggests that the culture of preventive care is poor among Nigerians in China.

On the perceived pattern of ill-health in the community, participants reported the occurrence of illnesses from communicable and non-communicable diseases. The most mentioned communicable disease was upper respiratory disease. Upper respiratory tract infection like the common cold was perceived to be caused by excessive exposure to Guangzhou weather during the winter seasons, especially among those who are homeless or rough sleeping. Describing how homelessness caused cold, a male participant explained that:

Majority of guys sleep on the streets; they sleep outside in this very cold weather; that is what makes boys get sick; when they don’t have accommodation. Guangzhou cold penetrates the bone. (IDI 1/Male/45 yrs.)

Other communicable diseases that caused illness in the community were malaria, typhoid and sexually transmitted infections.

Non-communicable diseases also contribute to ill-health in the community. The diseases mentioned ranged from obesity to haemorrhoids, renal diseases and hypertension. They attributed most non-communicable diseases to the type of foods available in the city. Specifically, there was a general perception that an economy of ‘fake foods’^3^ exists in Guangzhou. They perceived that fake foods are sold freely and openly in African-dominated sections. In places like Guangyuan Xi Lu, for instance, one male participant insisted that ‘…fake is much. They have fake [foods] like this apple. Or [alcoholic] drinks; the fake is too much. They are *jada jada*, that is fake’ (IDI 2/Male/47 years). A community leader similarly complained that the food market in Guangzhou was populated with organic produce, stating that, in China, ‘everything is hybrid; something you plant today and you harvest it tomorrow’ (KII 1/Male/48 years).

Mental health problem was not mentioned a lot, but it is important to highlight it because of how participant perceived the problem to be rooted in the day to day challenges and social pressures. Accordingly, a male participant explained that:

Some of us came here and become frustrated, no paper, no work, no house, no nothing… Some sold their land or borrowed money from different persons in different places to come to China. When they came here, they found themselves in a sorry situation and became frustrated. (IDI 3/Male/40 yrs.)

Another female participant (IDI 4/40 years) who hawks *moimoi*, a local dish from Nigeria, in markets around Guangyuan Xi Lu also reported that she was diagnosed with high blood pressure. Her health problem began in Nigeria after she separated from her partner with a baby on the way. She arrived in China pregnant and slept in an overcrowded apartment with other Nigerian male migrants. She was delivered of the birth in China, and both of them have lived in Guangzhou ever since—roughly 12 years at the interview time. The challenge for her, however, is that she was always afraid that the Chinese security agencies would arrest and deport her and her son on the ground of being undocumented. Although they were arrested and released on compassionate ground in the past, she was always afraid that they might not be lucky in the future. That constant worry over her child’s safety and the pain of separating from her partner many years ago remained the sources of anxiety for her in China.

Other sources of ill-health, though not prominent in participants’ narratives, were physical injuries resulting from accidents. In two reported incidents of physical injuries, Nigerian migrants were injured from a hit-and-run and during an attempt to evade visa checks.

### Experiences of Health Challenges in Everyday Life

This section is concerned with how the context at destination shapes the experiences of health challenges among Nigerians in Guangzhou. Four main themes had the most influence on how the participants were experiencing health problems: language, the cost of care, migration status and racism and discrimination.

#### Language Barrier

According to one survey of 233 Africans in Guangzhou, fewer than one-fifth of Africans were proficient speakers of Chinese (Zhou et al., 2016). The participants who had a more positive evaluation of Chinese health care spaces, hospitals and pharmacies, spoke a bit of Chinese or had friends who were available to serve as interpreters. However, language was a significant hindrance in assessing health care for many. On the one hand, the inability to handle conversational Chinese language prevented Nigerians from visiting the pharmacies or hospital to access care. Language problems made interaction with Chinese health personnel difficult or almost impossible. It also made the experience of hospitalisation unbearable, especially when it lasted for an extended period. Recalling his experience while on admission for a month, a participant reported that it was hard interacting with hospital staff (IDI 1/Male/45 years).

For minor health complaints, Nigerian migrants preferred to visit the pharmacy or traditional Chinese medicine stores. However, language was a barrier in these spaces as well as detailed below:

When you go, you tell them you have a headache, you do hand like this [cupped his head in his palms], they will give you another medicine. …You tell them that your head is paining you, they will give you [something else]. (IDI 5/Male/35 yrs.)

During consultations, language shaped how Chinese doctors interact with the participants. Precisely, good knowledge of Chinese made Chinese doctors more open to assisting, such that ‘…if the doctor realised that you could speak their language, they would be very happy but if you cannot communicate, there is no way they can help you’ (IDI 5/Male/35 years). Besides, limited language abilities to converse with migrant may lead to the withholding of diagnostic information from migrants unless a friend/family with language competence accompanied them to the hospital (IDI 6/Female/35 years).

#### ‘No Money’: a Context of Cost of Health Care

The cost of care was also shaping the experiences of health problems in Guangzhou. Some participant complained about ‘no money’ as an existential challenge. Having money refers to migrants’ ability to offset the direct costs associated with healthcare, and being socially positioned to meet healthcare-related expenses, including the mobilisation of supportive financial resources whenever the need arises. ‘No money’ prevents migrants from presenting illness in the hospital. Because many of them earn meagre income, there was pressure to save more money. A lot of them did not also have regular jobs and must be on the street from day to day to earn a living. Hence, spending on health was not considered a high priority (IDI 7/Male/26 years). Moreover, when hospitalised, ‘not having money’ is a source of worry and depression (IDI 8/Male/32 years). Even with those who have personal savings, a prolong hospitalisation drains savings quickly (IDI 1/Male/45 years).

More than lack of money, however, most of the participants perceived the cost of care as prohibitive. They perceived that Chinese doctors have a fetish for or ‘worship money’. The participants were often shocked when the cost of illnesses considered to be routine in Nigeria, e.g. common cold, run into several thousand of Renminbi. As one participant narrated:

I know of a guy that had cold which is a common thing here. They don’t treat cold [and] the guy spent close to RMB30,000 on it; if you change it to Nigerian money you know how much it is. He wasn’t treated [and] went back to Nigeria. He bought a tablet and it [cold] was off. (IDI 9/Male/30)

Consequently, some Nigerians prefer to delay their treatment until when they can return to Nigeria (IDI 10/Male/40 years).

However, a handful of participants felt that the cost of care was adequate and bearable. Those who viewed the cost of care favourably were mostly in the professional class or long-established Nigerians with thriving business ventures and offices in high-rise buildings in Guangzhou.

#### ‘Our People Are so Much Afraid of Police Catching Them’: Immigration Status

In a context where the host society has increasingly reformed immigration policy in ways that criminalised and imposed stiffer sanctions on ‘illegal’ entry, stay and work (Haugen, 2015), migration status assumes a critical role in everyday health challenges of migrants. Immigration status determined participants’ feeling and sense of personal safety when visiting the hospital. Feeling fear when visiting the hospital was prevalent in the community even though hospital staff rarely demand to know their status.

According to both community leaders and members, there was a prevailing misperception that made the hospital an unattractive place to seek care. They perceived hospitals as a space that should not be approached without valid immigration papers. In the words of a key informant:

…when you tell your [Nigerian] people to go to the hospitals they will be afraid, thinking that police will stand there to arrest them. …Our people are so much afraid of police catching them (KII 3/Male/34).

Even though hospitals do not demand to see passports, many undocumented Nigerians still avoid hospital spaces because of the fear of arrest.

However, there were situations when being undocumented predisposed migrants to stress and poor treatment in the hospital. In one hit-and-run case, a participant said the Chinese driver was not willing to offset the full medical bill unless the police verified his immigration status (IDI 2/Male/47 years). The participant recalls as follows:

Assuming I had paper, the Chinese driver will be responsible for everything. If a Chinese was knocked down in a hit-and-run, the victim will be on bed rest for as long as possible and there will be a settlement as well. But as far as you’re a foreigner, you cannot do anything. (IDI 2/Male/47)

Thus, despite not being a prerequisite for accessing health care, undocumented Nigerians were afraid of visiting hospitals. For some that ended up in the hospital, being undocumented can shape how one experiences access to health care.

#### Racism and Social Discrimination

The last theme discussed by many participants as shaping their experiences of health challenges in Guangzhou is racism and discrimination. A lot of those who have the first-hand experience of the hospitals in the city complained that, in comparison to Chinese people, they were treated differently. Some complained about experiencing poor treatment and discrimination because of their skin colour.

One dimension of discrimination is the lack of access to health insurance. Although married to a Chinese and had two children with her at the time the study, a participant complained that he was not legally allowed on the health insurance. As he said:

No foreigner is accepted in this [health insurance] system. The visa terms prohibit us from work [and] when you don’t work, you don’t have any mean of livelihood to pay for your health nor to be in a system that will allow you to enrol for health insurance. (LH 1/Male/45)

While married to Chinese, the problem of limited integration and exclusion was common with impact on how migrants experience health challenges.

On experiences of racism, there were accounts of racist behaviours during visits to the health facility. Based on her experience while visiting the hospital during the Ebola crisis of 2014, a female participant felt that a black person could be ‘unfortunate to have a racist as a [Chinese] doctor’ (IDI 6/Female 35). This period was also when the Chinese state stepped up checks at the major ports of entry to screen migrants visiting from Africa (Lin et al., 2015a, b; C. Liu, 2015).^4^

#### Supportive Networks, Community Structure and Medical Return Migration

Network and community structures helped Nigerian migrants in need of care to navigate the complications associated with the condition of being a foreigner and or undocumented. During routine hospital visits, a network of friends, which included Nigerians and Chinese acquaintances and associates, assist in identifying migrant-friendly hospitals and provide translation help to those lacking Chinese language competency. The community also advocate for better health-seeking behaviours by disseminating health information. ‘The leaders of the Nigerian Union advise Nigerians in China to go for medical check-ups’ (IDI 11/Male/37).

In emergencies and hospitalisation, friends were usually the first responders. They assist with hospital registration, meals and clothing. They make out-of-pocket payments to offset medical bills and escalate the news about severe illnesses to the community via social media platforms. Through a network of relations, those experiencing health problems mobilised large sums to deal with critical illness and pay for repatriation if required. When one migrant fell ill from renal failure ‘people supported [with] more than RMB 45,000’ (IDI 8/Male/32). Co-migrants also coordinate with the families of the sick—in Nigeria—in managing illness or planning repatriation.

However, community members sometimes become fatigued from helping. Prolonged hospitalisation, for instance, introduced a temporal dimension to the assistance that migrants render to one another. When this occurs, it becomes difficult for the sick migrant to continue to support their hospitalisation. A participant described his experience with a hospitalised friend as follows:

I’ve been in the hospital with him for at least one, two months, taking food there, I’d see him eating you know if you don’t have money here it’s also like Nigeria. [Later on] people cannot meet up they are not bringing money. (IDI 8/Male/32)

Part of the reason for this fatigue, explains a participant, is because in China, ‘everybody came to look for a means of survival’ (IDI 1/Male,45).

Moreover, when serious illnesses require repatriation, the role of the community expands. While fluid, omnipresent and active in fundraising, migrant community is distinguished for its roles in facilitating accelerated access to immigration clearance documents. The Nigerian community coordinates to assemble exit documents for three main categories of migrants with documentation problems. They include those with documents that are expired, lost/discarded and adopted/appropriated.^5^

Specifically, when medical repatriation becomes the last available choice for an undocumented but sick migrant, the Nigerian consulate in Guangzhou must issue a travel certificate (TC). Along with other relevant documents, the community heads would submit the TC to the Chinese authorities who give the final exit approval to the affected person.^6^

However, while those with expired identity document managed to process TC with minimal stress, Nigerians with lost/discarded and adopted/appropriated documents must prove and convince the Nigerian consulate about their Nigerian citizenship. At times, the verification leads to delays in the repatriation of chronically ill but undocumented Nigerians. In one case, such a delay worsened the health of the sick and led to further deterioration in which the migrants arrived in Nigeria and died after a few days.^7^

## Discussion

The article explored migration as social determinants of health challenges in everyday life of Nigerian migrants in Guangzhou, China. Although many of them perceived that they were in good health, community leaders believed that many Nigerian migrants have poor health-seeking behaviour, especially when it comes to accessing preventive care, including routine medical check-up. Nigerian migrants suffer from ill-health from communicable and non-communicable diseases, with language, cost of care, immigration status and racism and discrimination shaping their experiences of health issues. These micro-structural factors are known to be critical for the health of migrants in different settings, including China (Davis et al., 2016; Hasan et al., 2017; McLaughlin et al., 2015) and other Western countries (Biswas et al., 2011; Green et al., 2006; Ransford et al., 2010). Nevertheless, the present study uncovered some of the social processes behind the MSDH and how they potentially lead to a different understanding of migrant health.

On language, for instance, we found that knowing the Chinese language plays a critical role in coping with hospitalisation as a migrant. The sense of abandonment and isolation that migrants feel is most likely different from what obtains in one-off physician-migrant interactions that most studies tend to capture. Similarly, we showed that the perceived high cost of care was not only about the prohibitive medical charges for what migrants consider as routine. Instead, for our participants, the high cost of care was also about not having money, which is itself conditioned by the structure of labour market and the priorities of migrants in the use of their meagre income. More revealing, however, is the role of the migrant community as an essential element in the broader structures that shape how migrants experience health challenges.

Whether a destination is traditional or new determines the extent to which migrants would have access to and benefit from strong ties for mitigating health vulnerabilities (Derose et al., 2007). Derose et al. (2007) argue that unlike migrants residing in traditional migration spaces, those in new destinations are less likely to have strong social ties. Support from networks has a ‘stress-buffering effect,’ particularly in respect of helping migrants achieve ‘improved health and social functioning’ (Waldstein, 2008, p. 109). In this regard, the Nigerian community proved valuable.

However, we observe also that the capability of the community and network to alleviate challenging health experiences can be limited in many ways, depending on the complications involved in individual situations. For instance, there may be delays in processing documents required to facilitate medical returns, and community members can experience assistance fatigue due to the demands placed on them by their circumstances as (undocumented) migrants themselves.

Despite this, their coordination and engagement helped in navigating the bureaucracies critical for processing medical return migration between China and Nigeria. Medical return is part of the growing transnationalism in health (Davis et al., 2016; Green et al., 2006; Kane, 2012; Villa-Torres et al., 2017). However, previous studies were not attentive to how this return occurs, and the structural factor that shapes the process at destination. The present study showed that undocumented migrants experience difficulties in making a return migration when they fall chronically ill due to the constraints on-the-ground in Guangzhou city.

Being undocumented, sick and needing to return home creates a case of ‘involuntary return immobility’. The concept of involuntary return immobility builds on the notions of ‘involuntary immobility’ and ‘second state of immobility’ (Carling, 2002; Haugen, 2012). ‘Involuntary immobility’ explains the disjuncture between the aspiration to migrate and the ability of migrants to do so. In contrast, a ‘second state of immobility’ describes how migrants become spatially entrapped having escaped from their countries of origin to another country. While the desire of Nigerians to return was not aspirational—in a sense intended by Carling (2002)—they were nonetheless constrained to undertake a return for medical care. However, making a trip back to Nigeria was hampered by their inability to exit China legally. With the complexity in assembling documents, we see how migrants were forced to wait and become involuntarily immobile. This finding demonstrates that the processes of return medical migration are complicated by specific structural and institutional barriers, all of which point to the import of MSDH. The intermediate effect of undocumentedness in the process offers a fresh layer of constraint that studies have not documented.

It is worth noting that the experiences of participants examined in this article are similar and different from the experiences observed among migrant populations in similar and different situations and contexts. For example, Bork-Hüffer’s (2016, p. 50) comparative research showed that African migrants, including Nigerians, are similar to Chinese rural–urban migrants in terms of challenges such as ‘limited access to local health insurance schemes, low social status, discrimination and insecure legal status.’ However, Africans encounter language and racial barriers. Also, how discrimination, health insurance and legal status challenges are experienced can differ between Africans and Chinese internal migrants, owing to differences in citizenship, and their positionality to the local and international legal frameworks that shape access to health insurance and health services. Meanwhile, like Nigerians, Asian migrant workers in China also face barriers to healthcare, like structural, linguistic, financial and cultural kinds (Hall et al., 2019; Loganathan et al., 2020). But while they faced discrimination as well, migrants of Asian origin did not report racial discriminations. Thus, Nigerians in China may be closer to Africans outside China in terms of the range of healthcare-related challenges they face (see Mbanya, 2019). Nevertheless, while their differences are important, the similarities of experiences of migrants more generally reinforce the need to take a global approach to understanding and alleviating the health challenges that people on the move face across varied host country contexts.

Thus, examining MSDH challenges in everyday life has shown the necessity of paying attention to the processes that underlie the various factors that shape the experiences of health problems among migrants. Combining the MSDH with phenomenological approach also brings the structural factors closer to the realm of every day. The significance of this approach is potent in the light of the mantra of ‘leave no one behind’ in the 2030 Agenda for Sustainable Development. The Goal 3 of the Sustainable Development Goals, which aims to ‘ensure healthy lives and promote well-being for all at all ages’, is partly aimed at addressing the gap in the health needs of migrants (Tulloch et al. 2016; World Health Organisation, 2019).

## Strengths and Limitations of Study

A major strength of the article is that it grounds health challenges of Nigerian migrants in China in their everyday experiences and relationships as migrants of African origin in a Chinese city. Another strength lies in showing that although membership in community networks can help migrants in navigating health challenges, community’s capability to play such role is sometimes limited, hence weak in responding to health problems of migrants. This implies that as much as we wish to understand if individual migrants possess the capability to deal with their health-related issues, we must also assess if migrant communities are equally capable of serving the same purpose—and the barriers to doing so. The finding has implications for how community factor is deployed as a variable in migration and health research. Moreover, it highlights the importance of undocumentedness to medical return migration and the importance of language to migrants’ experience of hospitalisation. These evidences are shown to shape everyday health problems and contribute to the distinctive of their experiences when compared with migrants in China who may share similar precarity with African migrant population.

Conversely, an important limitation of the study is that it draws from the experiences of Nigerians in Guangzhou alone. Thus, the findings may not reflect the experiences of other African nationalities in the city—nor that of other Africans in other parts of China. Future studies can address this gap by incorporating the views of more diverse African nationalities towards generating a comprehensive evidence on how Africans in China experience health challenges as an everyday encounter.

## Conclusion

The study unveiled how migrants’ health challenges are shaped by the interdependencies of macro-structural factors and the everyday reality of being an African foreigner in China. Specifically, we showed that the culture of preventive care is poor among Nigerians in China and their experiences of health challenges by language, cost of care, immigration status and racial discrimination. While migrant networks and community structure provided critical assistance, especially in emergencies and hospitalisation, a sustained and or prolonged period of illness and hospitalisation may overstretch community resources and sources of support. In China, recognising health challenges as a phenomenon that is shaped by the very condition of migration, particularly the broader structures of the society, is crucial for tackling the SDH healthcare challenges among Africans.

## Data Availability

The data are not publicly available due to privacy or ethical restrictions. However, non-confidential research materials (e.g. interview guides and qualitative codebook) are available at https://urldefense.com/v3/__https://osf.io/wqyva__;!!N11eV2iwtfs!qWI4xTEbQIT_MLMvkewJKumjh1PJcbutNkmHoid03kpI6YrWAnrSBuEAkAIFgT9qeHWEA_Cn-Yezb4OCnua32AmV$.

## Footnotes

1 ‘Africans in China; as used in this article refers to people of African origin with citizenship of countries on the African continent.

2 This is self-reported information, and there was no way to verify it objectively. Moreover, some Nigerians carry valid passports of other African countries – Ghanaian, Nigerien, South African, Mozambican etc. – and have valid Chinese visas on them. There are other practices going on, including buying of passports of newly arrived or travelling outside Guangzhou to obtain visas; some enter with student visas by registering in schools outside Guangzhou while returning to live/work in Guangzhou, thereby remaining students perpetually. These are all avenues and bases to claim to have a valid visa, even though literature suggests that Nigerians in Guangzhou are predominantly undocumented (see Haugen, 2012).

3 Foods qualifying as ‘fake’ from the viewpoint of some Nigerians range from genetically modified foods to unapproved or adulterated foods, beverages, drinks and cigarettes.

4 Some Nigerians who arrived in China from Nigeria during the crisis said that they underwent special health screening at airports in Beijing and Guangzhou. However, the female participant did not have a travel history that may have warranted the suspiciousness with which the Chinese doctors treated her in the hospital.

5 From informal interactions and interviews, I gathered that some Nigerians discard their international passport once it expires while some others sell theirs with the visas still valid. However, others appropriated the nationality of other African countries by acquiring and using their passports to enter and reside in Guangzhou.

6 Before the Chinese authorities issue the exit approval, a migrant is subjected to a security interrogation.

7 There was a case in which a sick Nigerian migrant died after a day in Nigeria following a prolonged wait to obtain TC from the Nigerian consulate in Guangzhou.
